# Combined Nutraceutical Supplementation and Pulsed Electromagnetic Field Therapy Enhances Early Pain Reduction and Bone Callus Formation After Distal Radius Fracture: A Randomized Controlled Trial

**DOI:** 10.3390/nu18122010

**Published:** 2026-06-20

**Authors:** Dalila Scaturro, Sofia Tomasello, Marika Triscari Barberi, Giuseppe Lo Re, Giulia Letizia Mauro

**Affiliations:** 1 Precision Medicine in the Medical, Surgical and Critical Care Areas, University of Palermo, 90100 Palermo, Italy; giulia.letiziamauro@unipa.it; 2 Palermo Provincial Health Company, 90100 Palermo, Italy; sofia.tomasello@hotmail.it; 3 Biomedicine, Neuroscience and Advanced Diagnostics, University of Palermo, 90100 Palermo, Italy; marika.triscaribarberi@unipa.it (M.T.B.); giuseppe.lore@unipa.it (G.L.R.)

**Keywords:** rehabilitation, osteoporosis, fracture, distal radius epiphysis, pulsed electromagnetic fields, nutraceutical supplementation, methylsulfonylmethane

## Abstract

Background: Distal radius fractures (DRFs) are common fragility fractures often associated with underlying osteoporosis. Objective: To evaluate the effect of nutraceutical supplementation in addition to pulsed electromagnetic field (PEMF) therapy on pain and early fracture healing. Methods: Sixty female patients were randomized into two groups: Group A received PEMF therapy alone, while Group B received PEMF plus nutraceutical supplementation. The primary outcome was pain reduction (NRS). Secondary outcomes included biochemical markers and ultrasound-based callus formation. Results: At T1, Group B showed a trend toward greater pain reduction compared with Group A (mean difference −0.57; *p* = 0.007) and higher bone alkaline phosphatase levels (*p* = 0.0002). A higher proportion of patients reached minimal clinically important difference (MCID) in Group B (60% vs. 30%, *p* = 0.02). Conclusions: Nutraceutical supplementation in addition to PEMF was associated with improved short-term outcomes; however, due to the absence of a non-PEMF control group, the independent effect of PEMF cannot be determined.

## 1. Introduction

Distal radius fractures (DRFs) are among the most common orthopedic fractures, showing a bimodal distribution with a peak incidence in young adults following high-energy trauma and in peri- and post-menopausal women following low-energy injuries [1,2,3,4,5]. In older individuals, DRFs are frequently associated with osteoporosis and reduced bone mineral density, and their occurrence has been recognized as an early predictor of subsequent fragility fractures involving the vertebrae, humerus, and hip [6,7]. Functional outcomes, radiographic healing, and treatment approaches for DRFs have also been extensively investigated in both conservative and surgical settings [8,9,10]. Consequently, optimizing fracture healing and preventing future skeletal complications represent important clinical objectives.

The management of DRFs requires an integrated approach that combines fracture stabilization and rehabilitation strategies aimed at promoting functional recovery and bone repair [11,12,13,14,15,16]. Among adjunctive therapies, pulsed electromagnetic field (PEMF) treatment has attracted increasing attention because of its ability to modulate cellular signaling pathways involved in bone remodeling and osteogenesis. Experimental and clinical studies suggest that PEMFs may stimulate osteoblastic activity, enhance bone metabolism, and support fracture healing, although the available evidence remains heterogeneous and further high-quality studies are needed to clarify their clinical effectiveness [17,18]. Rehabilitation interventions, including physical therapy and therapeutic exercise, remain fundamental components of recovery and may act synergistically with biological therapies to improve outcomes after DRFs [19,20,21,22].

Nutritional status also plays a relevant role in bone metabolism and fracture healing. Several nutraceutical compounds, including vitamin D, vitamin K, magnesium, vitamin C, zinc, resveratrol, silicon, and methylsulfonylmethane, have been proposed as potential modulators of bone turnover and skeletal repair [23,24,25]. Among these compounds, methylsulfonylmethane (MSM) has attracted growing interest because of its potential anti-inflammatory and antioxidant properties, as well as its role in supporting collagen synthesis and connective tissue homeostasis. These biological mechanisms have been hypothesized to contribute to tissue repair and musculoskeletal recovery; however, robust clinical evidence supporting these effects in fracture healing is still limited, although clinical evidence in fracture healing remains limited. However, contemporary evidence remains inconsistent and, in some cases, controversial. For example, large randomized trials have shown that vitamin D supplementation alone does not significantly reduce fracture risk, whereas potential benefits may depend on combined supplementation strategies and patient-specific characteristics [26]. Therefore, the effectiveness of multimodal nutraceutical supplementation in the context of fracture healing remains incompletely understood.

In recent years, musculoskeletal ultrasound has emerged as a useful tool for monitoring fracture healing. In addition to being safe, repeatable, and free of ionizing radiation, ultrasound can identify periosteal reactions and early callus formation before these changes become evident on conventional radiographs, providing valuable information regarding the progression of bone repair [27,28,29,30,31,32,33,34]. Furthermore, ultrasound allows for simultaneous assessment of surrounding soft tissues and local inflammatory changes, making it particularly suitable for the early follow-up of fracture healing.

The evaluation of bone metabolism and fracture healing is particularly relevant in patients at risk of osteoporosis, as altered bone turnover may influence the reparative process and predispose patients to future fragility fractures [34,35]. Moreover, inadequate recovery after DRF may be associated with complications such as complex regional pain syndrome (CRPS), a disabling condition characterized by persistent pain and functional impairment [36,37].

The aim of the present randomized controlled trial was to evaluate the effectiveness of multimodal nutraceutical supplementation in patients with recent distal radius fractures receiving PEMF therapy. The primary outcome was the change in pain intensity measured by the Numeric Rating Scale (NRS). Secondary outcomes included biochemical markers of bone turnover, ultrasound-based assessment of callus formation, and the incidence of complex regional pain syndrome (CRPS).

To the best of our knowledge, this is the first randomized controlled trial investigating the combined effects of PEMF therapy and multimodal nutraceutical supplementation on early clinical, biochemical, and ultrasound outcomes following distal radius fracture.

We hypothesized that the addition of multimodal nutraceutical supplementation to PEMF therapy would result in greater pain reduction, enhanced bone turnover activity, and earlier ultrasound evidence of callus formation compared with PEMF therapy alone.

## 2. Materials and Methods

### 2.1. Study Design

At the Functional Recovery and Rehabilitation Unit of the “Paolo Giaccone” University Hospital in Palermo, in collaboration with the Radiodiagnostics Unit of the “Paolo Giaccone” University Hospital in Palermo, we conducted a single-center, non-blinded randomized controlled trial (RCT) involving a population of patients with a recent distal radius fracture. The study was specifically designed to investigate early-phase fracture healing rather than long-term metabolic equilibrium.

The non-blinded design was adopted due to the practical difficulties in masking heterogeneous interventions such as oral nutraceutical supplementation and PEMF therapy, as well as to ensure adequate treatment adherence and management. The potential risk of bias was partially mitigated by the use of objective outcomes (biochemical markers and ultrasound assessment). However, the lack of blinding represents a limitation, particularly for subjective outcomes such as pain.

The study was conducted between March 2024 and December 2025. For data collection, we included a consecutive series of patients who were referred to the Functional Recovery and Rehabilitation Unit of the A.O.U.P. “Paolo Giaccone” of Palermo between April 2024 and November 2025 for a physiatrist evaluation.

The study received approval from the Local Ethics Committee “Palermo 1” (Approval No. 8/2024) and was conducted in accordance with the Declaration of Helsinki. All information and data were handled according to Good Clinical Practice (GCP) guidelines.

The nutraceutical used in the study is duly registered in the Italian Ministry of Health register of food supplements. The compound was produced according to Good Manufacturing Practice (GMP) standards in order to ensure continuous quality control; product compliance was also verified prior to administration.

All participants signed written informed consent before inclusion. The study was conducted in accordance with the CONSORT guidelines for randomized controlled trials (RCTs) and was also registered on ClinicalTrials.gov (NCT07352501, 19 December 2025).

### 2.2. Participants

The inclusion criteria were as follows: female sex, age between 45 and 55 years, diagnosis of a recent distal radius fracture with radiographic evidence of the fracture line, Numeric Rating Scale (NRS) score at T0 ≥ 4, and written informed consent.

The following subjects were excluded from the study: male patients, pregnant women, patients with neoplasms, carriers of pacemakers or other implantable devices, individuals presenting skin lesions and/or local infections, those with tendon injuries, patients suffering from cervical myelopathy, and individuals with contraindications and/or allergies to the active components of the nutraceutical supplementation.

Using our hospital database, we recruited a consecutive series of patients meeting the above-mentioned criteria who subsequently underwent rehabilitation treatment.

### 2.3. Interventions

A total of 60 patients with a recent distal radius fracture (DRF) were recruited and randomly allocated in a 1:1 ratio into two groups using a computer-generated random number system. The allocation was therefore completely random and performed without considering baseline (T0) data.

Group A, consisting of 30 patients, received 30 sessions of Pulsed Electromagnetic Fields (PEMF) therapy (9 h per day for one month).

Group B, consisting of 30 patients, underwent 30 PEMF sessions with the same timing and protocol as Group A and additionally received a daily oral nutraceutical supplementation for 30 days, containing methylsulfonylmethane, magnesium, vitamin C, vitamin E, vitamin B12, vitamin K2, silicon, resveratrol, zinc, and vitamin D3.

Patients in both groups were allowed to use paracetamol 1000 mg as needed, up to a maximum of three times per day.

### 2.4. Group A (PEMF)

Participants in Group A attended the outpatient clinics of our department and were received by a team consisting of a physiatrist and a physiotherapist, who explained the timing and methods of use of the PEMF devices, so that they could use them for 9 h per day at home, preferably during night-time hours.

#### PEMF Protocol

We used the Magneto 4 Chinesport device (Chinesport S.p.A., Udine, Italy), compatible with the presence of a plaster cast, with the following settings: Frequency: 15–50 Hz; Magnetic field intensity: 30–100 Gauss; Waveform: low-frequency pulsed; Mode: continuous; Use of magnetic bands; Direct positioning on the fracture site; Single session duration: 60 min; Frequency: 1 session per day.

The PEMF protocol adopted in this study was defined based on parameters commonly reported in the literature for the treatment of bone fractures, particularly in terms of low-frequency stimulation (15–50 Hz) and magnetic field intensity (30–100 Gauss), which have been associated with stimulation of osteoblastic activity and promotion of bone remodeling processes [17,18]. The daily duration of exposure (up to 9 h/day) and total treatment period (30 days) were selected in accordance with previous clinical and experimental studies suggesting that prolonged and repeated exposure enhances the biological effects of PEMF on bone healing [17,18,19].

To ensure treatment standardization, all patients received detailed instructions from trained healthcare professionals regarding device use, positioning, and daily duration. The devices were used under home-based conditions following a structured protocol, and patients were periodically monitored during outpatient visits to verify adherence. Compliance was reinforced through verbal instructions and follow-up checks.

The PEMF device used (Magneto 4, Chinesport, Udine, Italy) is a commercially available, certified medical device compliant with European regulatory standards (CE marking). The device allows for reproducible delivery of pulsed electromagnetic fields with predefined and adjustable parameters, ensuring consistency of treatment across participants.

### 2.5. Group B (NS)

Participants in Group B performed the same PEMF sessions as Group A with the same timing and modalities, and additionally received daily nutraceutical supplementation for 30 days. The supplement contained methylsulfonylmethane (1000 mg), vitamin E (30 mg), vitamin B12 (500 µg), vitamin B2 (12.5 mg), vitamin B6 (5 mg), silicon (8.5 mg), resveratrol (25 mg), vitamin D (50 µg), vitamin K (90 µg), magnesium (225 mg), vitamin C (500 mg), zinc (7.5 mg), copper (1 mg), and folate (200 µg).

Patients were asked to take the supplement every day at the same time and on an empty stomach in order to optimize absorption. They were also advised to store the product at a temperature below 25 °C in a cool and dry place, away from sources of light and heat. The compound was gluten-free and lactose-free, thus ensuring safe administration.

### 2.6. Clinical Evaluation

Demographic and clinical information were obtained from the patients’ medical records. The following assessments were conducted at three different time points: at baseline (T0), a two-view wrist radiographic examination was performed and, after blood sampling and 24-h urine collection, the levels of Vitamin D, Calcium, Phosphorus, Magnesium, CTX, total alkaline phosphatase and bone isoenzyme, Parathyroid hormone, 24-h calciuria and phosphaturia were measured for the evaluation of bone turnover; at 15 days after the start of treatment (T1), laboratory tests were repeated and an ultrasound examination of the wrist and hand was performed for early evaluation of bone callus formation; finally, after 30 days of follow-up (T2), a control radiographic examination was performed and the “Budapest Criteria” were analyzed for the early diagnosis of Complex Regional Pain Syndrome. Pain intensity was considered at each evaluation using the Numeric Rating Scale (NRS), an 11-point scale ranging from 0 (no pain) to 10 (the worst pain imaginable).

### 2.7. Ultrasound Criteria

Ultrasound examination (US) was used as an early assessment tool for the evaluation of bone callus formation and the condition of peri-fracture soft tissues [31,32].

To minimize operator-dependent variability, all ultrasound examinations were performed by the same experienced radiologist with specific expertise in musculoskeletal imaging, following a standardized acquisition protocol. The evaluation was conducted using predefined morphological and vascular criteria derived from previously published studies on ultrasound assessment of fracture healing [31,32,33,34].

The semi-quantitative scoring system adopted in this study was based on the integrated assessment of multiple ultrasound parameters (cortical continuity, callus morphology, echogenicity, and vascularization), which have been described as indicators of different stages of bone repair. Although this approach has been used in previous studies, it does not represent a fully validated scoring system and may be subject to a degree of observer-dependent interpretation.

To reduce subjectivity, all assessments were performed under standardized conditions, and the same operator evaluated all patients, ensuring internal consistency. However, formal intra- and inter-observer reliability analyses were not performed and should be considered in future studies.

The ultrasound investigation was performed at T1 (15 days after the traumatic event) by an experienced radiologist at the Imaging Diagnostics Unit of the Paolo Giaccone University Hospital in Palermo, using a high-resolution ultrasound device with a high-frequency linear probe (10–18 MHz).

The patient was examined in a seated position, with the upper limb supported and the wrist in a neutral position. The examination was conducted in both longitudinal and transverse planes, systematically exploring the bony cortex of the distal radius epiphysis, the fracture line site, and the surrounding soft tissues.

The main ultrasound parameters analyzed included:

- Continuity and regularity of the bone cortex, with particular attention to the presence of discontinuities or irregularities of the cortical profile;
- Presence of early bone callus, identified as a hyperechoic or moderately hyperechoic periosteal area, possibly associated with posterior acoustic shadowing;
- Thickness and morphology of the bone callus, qualitatively assessed;
- Presence of peri-fracture soft tissue edema, defined as increased thickness and hypoechogenicity of the subcutaneous tissues;
- Possible joint effusion or signs of tendon involvement;
- Power Doppler evaluation, when necessary, to highlight increased local vascularization, indicative of reparative activity or inflammatory processes [34,35].

The presence of an ultrasonographically detectable bone callus was considered an indicator of the initiation of the bone consolidation process [36]. Ultrasound also allowed for a dynamic and non-invasive evaluation of soft tissues, proving particularly useful for monitoring edema and for the early identification of signs compatible with algodystrophic evolution.

Ultrasound examination was chosen because it is a safe, repeatable technique, free of ionizing radiation, and sensitive in the early detection of bone and periosteal changes, allowing for an earlier prognostic assessment compared with conventional radiographic examination [34,37].

### 2.8. Ultrasound Evaluation

The ultrasound evaluation of bone callus was conducted using a semi-quantitative approach, based on the integrated analysis of six morphological and vascular ultrasound parameters, considered representative of the different phases of the bone repair process [31,32,33,34,35,36,37,38].

Each parameter was assessed according to a progressive severity gradient (absent, moderate, marked), allowing for an overall classification of the stage of healing.

First, the continuity of the bone cortex was analyzed by observing the profile of the distal radius in both longitudinal and transverse planes. The persistence of a clear cortical discontinuity was interpreted as an expression of an early reparative process, whereas the presence of periosteal irregularities with partial reconstruction of the cortical profile suggested the initiation of bone remodeling. A nearly continuous reconstruction of the cortex, although still irregular, was considered indicative of early consolidation.

Subsequently, the presence and distribution of the periosteal bone callus were evaluated, identified ultrasonographically as hyperechoic tissue adjacent to the fracture site. The simple appearance of thin hyperechoic areas was considered an initial sign of callus formation, whereas a more voluminous and well-defined structure was interpreted as evidence of a more advanced reparative response.

Another element of evaluation was the thickness of the bone callus, estimated qualitatively. A thin and poorly organized callus was associated with the early stages of healing, whereas an increase in thickness with greater structural definition was considered indicative of progressive maturation of the newly formed bone tissue.

Particular attention was given to the echogenicity of the bone callus, a parameter related to the degree of mineralization. A predominantly hypoechoic callus was interpreted as early fibrous or cartilaginous tissue; the presence of a mixed hypo–hyperechoic pattern was considered characteristic of intermediate phases, whereas predominant hyperechogenicity was associated with greater mineralization of the callus [32,33,34,35,36,37,38,39].

The structural integration between the bone callus and the cortex was also evaluated by observing the morphological continuity between the newly formed tissue and the native bone. A disorganized callus separated from the cortex was considered a sign of incomplete integration, whereas progressive structural fusion was interpreted as indicative of effective bone remodeling [30].

Finally, the biological activity of the callus was studied using Color Doppler, evaluating the presence and intensity of periosteal and intralesional vascular signals. The absence of a Doppler signal was associated with reduced metabolic activity; a modest and localized signal was considered physiological in the early stages, whereas more intense and diffuse vascularization was interpreted as evidence of high reparative activity [31,32].

The integration of the six parameters allowed for the classification of the healing process into progressive stages, from an initial reparative response phase to advanced early consolidation. This descriptive approach enabled a dynamic, repeatable, and clinically meaningful ultrasound evaluation, facilitating correlation with the clinical, laboratory, and functional data of the study (see Table 1).

### 2.9. Statistical Analysis

Data collection was performed using a spreadsheet (Microsoft Excel, version 16.58). The study was conducted following the CONSORT guidelines for randomized controlled trials (RCTs). A priori sample size calculation was performed based on the primary outcome of pain reduction measured by the Numeric Rating Scale (NRS). The estimation was conducted using G*Power software (version 3.1.9.4), assuming a between-group mean difference of 1.5 points on the NRS, with a standard deviation of 2.0, corresponding to a moderate effect size (Cohen’s d ≈ 0.75). A two-tailed α level of 0.01 and a statistical power (1 − β) of 0.80 were set. Based on these assumptions, the required sample size was 53 participants. To account for potential dropouts, a total of 60 patients were included in the study. Changes in scores across the different evaluation scales were analyzed using the standard error of measurement (SEM) distribution-based method to determine clinical improvement. From this method, the minimal clinically important difference (MCID) was calculated for each instrument, defined as the minimum difference perceived as beneficial by both patients and clinicians. Based on the SEM, a change of 2.8 points in the NRS corresponded to the MCID. It should be noted that the exact value of the MCID is not fixed and depends on the method used to calculate the change in score; generally, a reduction of 2 points or 30% is considered clinically meaningful. The MCID analysis was intended to identify individual responders and should not be interpreted as equivalent to mean between-group differences. Therefore, it is possible for a substantial proportion of patients to achieve clinically meaningful improvement even when the average group change remains below the MCID threshold. The normality of the collected data was assessed using the Shapiro–Wilk test. Continuous variables were expressed as means and standard deviations, while categorical variables were reported as absolute numbers and percentages. Given the repeated-measures design, particular attention was paid to the evaluation of changes over time and between groups. Although pairwise comparisons were initially used to explore differences at specific time points (e.g., *t*-tests), the interpretation of the results was guided by the overall group × time framework, in order to account for the longitudinal nature of the data and to reduce the risk of type I error. To compare the different treatments, we applied Tukey’s honestly significant difference (HSD) procedure, which facilitates pairwise comparisons within ANOVA data. The F statistic indicates whether an overall difference exists among the sample means, while Tukey’s HSD test identifies which pairs of values, if any, differ significantly. Categorical variables were compared using the chi-square test or Fisher’s exact test, as appropriate. Statistical analysis was performed using the R statistical software (R Core Team, Vienna, Austria, 2021). Results with *p* ≤ 0.05 were considered statistically significant.

## 3. Results

A total of 67 patients with a recent distal radius fracture were initially screened. Of these, 4 patients did not meet the inclusion criteria, and 3 met exclusion criteria; therefore, 60 patients were included in the study and randomly allocated into two groups of equal size.

Baseline demographic and clinical characteristics are reported in Table 2. The two groups were comparable at baseline, with no statistically significant differences observed for age, pain intensity (NRS), biochemical parameters, or prevalence of osteoporosis, confirming the homogeneity of the sample.

The main clinically relevant outcomes at baseline (T0) and after 15 days of treatment (T1) are summarized in Table 3. Both groups showed a reduction in pain intensity over time; however, patients receiving PEMF combined with nutraceutical supplementation achieved significantly lower NRS scores at T1 compared with the PEMF-only group (*p* = 0.007). Similarly, bone alkaline phosphatase (BALP), a marker of osteoblastic activity and bone formation, was significantly higher in the combined treatment group at T1 (*p* = 0.0002). No significant between-group differences were observed for calcium, magnesium, or CTX levels at the same time point.

The clinical relevance of these findings was further supported by the analysis of the minimal clinically important difference (MCID). A significantly higher proportion of patients in the PEMF + nutraceutical supplementation group achieved clinically meaningful pain reduction compared with the PEMF group (60% vs. 30%, *p* = 0.02) (Table 4). This analysis reflects the proportion of individual patients achieving the predefined MCID threshold and should not be interpreted as equivalent to the mean change observed at the group level.

Ultrasound evaluation (Table 5) demonstrated a significantly higher proportion of patients with organized callus formation in the PEMF + nutraceutical supplementation group (54% vs. 13%, *p* < 0.001), whereas the absence of callus was more frequent in the PEMF group. These findings suggest a more advanced early reparative process in patients receiving the combined treatment.

The incidence of CRPS was low in both groups, and no statistically significant differences were observed (Table 6). Given the limited number of events, these results should be interpreted as descriptive.

To provide a more integrated interpretation of treatment effects over time, repeated-measures and mixed-design ANOVA analyses were performed. Detailed repeated-measures ANOVA results are reported in Supplementary Tables S1–S3.

For pain intensity (NRS), no significant group × time interaction was observed (partial eta squared = 0.054), indicating a comparable temporal reduction in both groups. However, at T1, the between-group mean difference was −0.57 points (95% CI −0.98 to −0.16), with a moderate effect size (Cohen’s d = 0.71), suggesting a clinically relevant advantage of the combined treatment.

In contrast, a significant group × time interaction was observed for bone alkaline phosphatase (BALP) (partial eta squared = 0.527), indicating a markedly greater increase over time in the PEMF + nutraceutical supplementation group. At T1, the between-group mean difference was 5.13 U/L (95% CI 2.48 to 7.79), with a large effect size (Cohen’s d = 1.00), confirming the magnitude of this effect.

Significant interaction effects of small-to-moderate magnitude were also observed for calcium (partial eta squared = 0.124), magnesium (partial eta squared = 0.137), and CTX (partial eta squared = 0.119), whereas no significant interactions were found for vitamin D, phosphorus, total alkaline phosphatase, calciuria, or phosphaturia. Overall, these findings indicate a more pronounced and consistent biological response in the combined treatment group.

## 4. Discussion

The present randomized controlled trial investigated the effects of combined nutraceutical supplementation and pulsed electromagnetic field (PEMF) therapy on early clinical and biological outcomes following distal radius fractures. Overall, our findings suggest that the addition of nutraceutical supplementation to PEMF therapy is associated with improved short-term outcomes, particularly in terms of pain reduction, bone turnover markers, and early callus formation.

From a clinical perspective, both groups showed a reduction in pain over time; however, patients receiving the combined treatment demonstrated a greater proportion of clinically meaningful improvement, as reflected by the higher percentage of individuals achieving the MCID. Although the repeated-measures analysis did not show a statistically significant group × time interaction for NRS, the observed moderate effect size and the between-group difference at T1 may suggest a potential additive benefit of nutraceutical supplementation in the early phase of recovery. It should be noted that the absolute between-group difference in NRS score at T1 was relatively modest (−0.57 points). Therefore, the statistical significance of this finding should not be interpreted as evidence of a large clinical effect at the population level. However, the higher proportion of patients achieving the MCID in the combined treatment group suggests that the intervention may have provided a clinically meaningful benefit for a substantial subset of participants. In addition, the observed moderate effect size supports the possibility of a relevant treatment effect despite the relatively small mean difference. Nevertheless, these findings should be interpreted cautiously and confirmed in larger studies with longer follow-up periods to determine their practical implications for routine clinical care. This finding is clinically relevant, as early pain reduction has been associated with improved adherence to rehabilitation and better functional outcomes [21].

A more consistent pattern emerged for biochemical markers of bone metabolism. In particular, the significant and large group × time interaction observed for bone alkaline phosphatase (BALP), together with moderate effects for calcium, magnesium, and CTX, indicates a more pronounced biological response in the group receiving nutraceutical supplementation. BALP is a well-recognized marker of osteoblastic activity and bone formation, and its increase may reflect an enhanced early reparative process. These findings are in line with current evidence suggesting that nutritional status and micronutrient availability play a modulatory role in bone remodeling and fracture healing, although the magnitude and consistency of these effects remain debated [40].

The ultrasound findings further support this interpretation. The higher proportion of patients with organized callus formation in the supplemented group suggests a more advanced stage of early bone repair. However, these findings should be interpreted with caution. The ultrasound assessment was performed only 15 days after fracture occurrence and therefore reflects an early phase of the reparative process rather than definitive bone consolidation. Consequently, the presence of a more organized callus at this stage cannot be assumed to predict superior long-term healing, fracture union, or functional recovery. Since no long-term radiographic or functional outcomes were collected, it remains unclear whether the observed early biological advantages translate into sustained clinical benefits. Future studies with extended follow-up are needed to determine the prognostic significance of these early ultrasound findings. Ultrasound has been increasingly recognized as a sensitive tool for the early detection of callus formation, often identifying reparative changes earlier than conventional radiography [31]. However, given the semi-quantitative nature of the assessment and the absence of formal reliability testing, these findings should be interpreted with caution and considered exploratory.

The role of PEMF therapy in fracture healing remains controversial. Experimental studies suggest that PEMF may influence osteogenesis through modulation of cellular signaling pathways and stimulation of osteoblastic activity [18]. However, clinical evidence is heterogeneous, with systematic reviews reporting inconsistent results and limited high-quality data.

The interpretation of nutraceutical supplementation is similarly complex. While several components included in the formulation—such as vitamin D, vitamin K, magnesium, and vitamin C—are known to be involved in bone metabolism, current evidence does not support a consistent or universal benefit of supplementation. For instance, large randomized trials have shown that vitamin D supplementation alone does not significantly reduce fracture risk [30]. Similarly, recent reviews emphasize that the effects of micronutrient supplementation are context-dependent and influenced by baseline nutritional status, combination therapies, and patient characteristics [24]. Therefore, the positive findings observed in our study should be interpreted as preliminary and specific to the combined formulation and clinical context investigated.

Regarding complications, the incidence of CRPS was low and not significantly different between groups. Given the limited number of events, no definitive conclusions can be drawn, and this outcome should be considered descriptive.

Several limitations should be acknowledged. First, the absence of a non-PEMF control group does not allow for independent evaluation of the effect of PEMF or a clear assessment of potential synergistic effects. Second, the relatively small sample size and short follow-up period limit the generalizability of the findings and preclude conclusions on long-term outcomes. Third, the open-label design may have introduced bias, particularly for subjective outcomes such as pain. In addition, analgesic consumption was not systematically quantified. Future studies should adopt placebo-controlled and assessor-blinded designs to minimize performance and detection bias. Finally, the semi-quantitative ultrasound assessment, although standardized, may be subject to operator-dependent variability, and no formal intra- or inter-observer reliability analysis was performed.

Although all ultrasound examinations were performed by the same experienced radiologist using a standardized protocol, the absence of formal reproducibility analyses limits the ability to quantify measurement reliability. Consequently, some degree of observer-dependent variability cannot be excluded, particularly in the interpretation of semi-quantitative parameters such as callus morphology, cortical integration, and vascularization. Therefore, the ultrasound findings should be interpreted with appropriate caution and considered supportive rather than definitive evidence of enhanced bone repair. Future studies should incorporate intra- and inter-observer reliability assessments and blinded image evaluation to improve the methodological robustness of ultrasound-based outcomes.

An additional important limitation is the absence of a non-PEMF control group. Because all participants received PEMF therapy, the present study was designed to evaluate only the potential additional benefit of nutraceutical supplementation within a PEMF-treated population. Consequently, the independent contribution of PEMF therapy to the observed clinical and biological outcomes cannot be determined, nor can potential synergistic effects between PEMF and nutraceutical supplementation be conclusively established. Therefore, the findings should not be interpreted as evidence of the efficacy of PEMF itself, and future controlled trials including a non-PEMF comparator are required to clarify the specific role of each intervention.

Despite these limitations, this study provides preliminary evidence suggesting that nutraceutical supplementation may enhance early clinical and biological recovery in patients undergoing PEMF therapy for distal radius fractures. Future randomized controlled trials with larger sample sizes, longer follow-up, inclusion of appropriate control groups, and more robust statistical models (e.g., mixed-effects models) are needed to confirm these findings and better define the role of combined therapeutic strategies in fracture management.

## 5. Conclusions

In conclusion, the findings of the present study suggest that nutraceutical supplementation in patients undergoing PEMF therapy may be associated with improved early clinical and biological outcomes following distal radius fractures. In particular, the combined approach was associated with a trend toward greater pain reduction, a higher proportion of patients achieving clinically meaningful improvement, and a more pronounced early bone reparative response.

However, these findings should be interpreted with caution due to the methodological limitations of the study, including the lack of a non-PEMF control group, the relatively small sample size, and the short follow-up period. Therefore, nutraceutical supplementation may represent a promising and potentially useful adjunctive strategy, but further well-designed randomized controlled trials are needed to confirm these results and to clarify its role in clinical practice. Importantly, because all participants received PEMF therapy, the present study does not allow conclusions regarding the independent efficacy of PEMF. The observed findings should therefore be interpreted as the potential additional effect of nutraceutical supplementation in patients undergoing PEMF treatment.

## Figures and Tables

**Table 1:** Semi-quantitative ultrasound criteria used for the assessment of early bone callus formation and fracture healing progression.

Ultrasound Parameter	Absent/Initial	Moderate/Intermediate	Marked/Advanced
**Cortical bone continuity**	Clear cortical discontinuity, evident fracture margins	Periosteal irregularities with partial reconstruction of the cortical profile	Cortical profile almost continuous
**Presence and distribution of periosteal callus**	Absence or thin periosteal hyperechoic areas	Evident periosteal callus, moderately extended	Voluminous, well-defined and widespread callus
**Bone callus thickness**	Thin, poorly organized callus	Moderate increase in thickness with initial organization	Thick and structurally defined callus
**Callus echogenicity**	Predominantly hypoechoic (fibrous/cartilaginous tissue)	Mixed hypo–hyperechoic pattern	Predominantly hyperechoic
**Callus–cortex integration**	Disorganized callus separated from the cortex	Initial continuity	Evident structural fusion and progressive remodeling
**Vascularization (Color Doppler)**	Absence of vascular signal	Modest and localized signal	Intense and diffuse vascularization, high reparative activity

**Table 2:** Baseline characteristics of the study population.

	Group A (PEMF) (*n* = 30)	Group B (PEMF + NS) (*n* = 30)	*p*-Value
Age (years)	49.00 ± 2.68	49.07 ± 2.58	>0.05
NRS	5.60 ± 1.10	5.43 ± 1.01	>0.05
25(OH)D (ng/mL)	29.48 ± 6.20	29.74 ± 5.98	>0.05
Calcium (mg/dL)	9.17 ± 0.70	9.20 ± 0.77	>0.05
Phosphorus (mg/dL)	3.44 ± 0.67	3.45 ± 0.63	>0.05
Magnesium (mg/dL)	1.96 ± 0.21	1.92 ± 0.19	>0.05
CTX (ng/mL)	0.74 ± 0.38	0.75 ± 0.37	>0.05
ALP (U/L)	84.66 ± 24.75	84.43 ± 23.51	>0.05
BALP (U/L)	16.90 ± 6.30	16.86 ± 6.07	>0.05
Calciuria (mg/24 h)	179.73 ± 39.86	179.53 ± 39.41	>0.05
Phosphaturia (mg/24 h)	0.86 ± 0.26	0.85 ± 0.22	>0.05
Osteoporosis, *n* (%)	1 (3.3)	2 (6.7)	1.000

**Table 3:** Main clinical and biochemical outcomes.

	Group A T0	Group A T1	Group B T0	Group B T1	*p*-Value at T1
NRS	5.60 ± 1.10	4.03 ± 0.85	5.43 ± 1.01	3.46 ± 0.73	0.007
BALP (U/L)	16.90 ± 6.30	18.56 ± 5.83	16.86 ± 6.07	23.70 ± 4.32	<0.001
Calcium (mg/dL)	9.17 ± 0.70	9.44 ± 0.72	9.20 ± 0.77	9.63 ± 0.74	0.330
Magnesium (mg/dL)	1.96 ± 0.21	2.03 ± 0.16	1.92 ± 0.19	2.10 ± 0.18	0.130
CTX (ng/mL)	0.74 ± 0.38	0.89 ± 0.37	0.75 ± 0.37	0.98 ± 0.36	0.320

Abbreviations: NRS, Numeric Rating Scale; BALP, bone alkaline phosphatase; CTX, C-terminal telopeptide of type I collagen.

**Table 4:** Change in pain intensity and MCID achievement.

Group	ΔNRS (T1 − T0)	Patients Achieving MCID, *n* (%)	*p*-Value
PEMF	−1.57 ± 0.62	9 (30.0)	
PEMF + NS	−1.97 ± 0.58	18 (60.0)	0.020

**Table 5:** Ultrasound assessment of callus formation at T1.

Ultrasound Outcome	Group A *n* (%)	Group B *n* (%)	*p*-Value
No callus	12 (40.0)	4 (13.3)	
Initial callus	14 (46.7)	10 (33.3)	
Organized callus	4 (13.3)	16 (53.3)	<0.001

**Table 6:** Incidence of CRPS according to Budapest criteria.

Group	CRPS Positive *n* (%)	CRPS Negative *n* (%)	*p*-Value
PEMF	2 (6.7)	28 (93.3)	
PEMF + NS	1 (3.3)	29 (96.7)	0.554

## Data Availability

The original contributions presented in the study are included in the article/Supplementary Material, further inquiries can be directed to the corresponding author.

## Supplementary Materials

The following supporting information can be downloaded at: https://www.mdpi.com/article/10.3390/nu18122010/s1, Table S1. Repeated-measures ANOVA summary of treatment effects from baseline (T0) to 15 days (T1). Table S2. Mixed-design ANOVA results. Table S3. Effect sizes and confidence intervals for significant outcomes.

## Abbreviations

**Abbreviation**	**Full Term**
DRF	Distal radius fracture
NRS	Numeric Rating Scale
MCID	Minimal clinically important difference
PEMF	Pulsed electromagnetic fields
NS	Nutraceutical supplementation
CTX	C-terminal telopeptide of type I collagen
ALP	Alkaline phosphatase
BALP	Bone alkaline phosphatase
CRPS	Complex regional pain syndrome
US	Ultrasound
CI	Confidence interval
η^2^	Partial eta squared
