# Supplementary material for: Combined Nutraceutical Supplementation and Pulsed Electromagnetic Field Therapy Enhances Early Pain Reduction and Bone Callus Formation After Distal Radius Fracture: A Randomized Controlled Trial
Source: Nutrients. 2026 Jun 20;18(12):2010. doi: 10.3390/nu18122010 (PMC13305784; doi:10.3390/nu18122010)
Supplement: Supplementary file 1 [file nutrients-18-02010-s001.zip › nutrients-4343987-supplementary.pdf]

Supplementary Table S1. Repeated-measures ANOVA summary of treatment effects from baseline (T0) to 15 days (T1).

Variable	Group A T0	Group A T1	Change	Group B T0	Group B T1	Change	F interaction (1,58)	p-value	Partial η²	Interpretation
NRS	5.600	4.033	−1.567	5.433	3.467	−1.967	3.288	0.075	0.054	No significant interaction
25(OH)D	29.480	32.340	2.860	29.740	32.593	2.853	0.000	0.987	0.000	No significant interaction
Calcium	9.173	9.443	0.270	9.203	9.627	0.423	8.193	0.006	0.124	Significant interaction
Phosphorus	3.443	3.643	0.200	3.450	3.763	0.313	3.351	0.072	0.055	No significant interaction
Magnesium	1.957	2.037	0.080	1.920	2.103	0.183	9.201	0.004	0.137	Significant interaction
CTX	0.748	0.889	0.141	0.751	0.982	0.232	7.830	0.007	0.119	Significant interaction
ALP	84.667	95.000	10.333	84.433	93.533	9.100	0.233	0.631	0.004	No significant interaction
BALP	16.900	18.567	1.667	16.867	23.700	6.833	64.721	<0.001	0.527	Significant interaction
Calciuria	179.733	188.167	8.433	179.533	187.967	8.433	0.000	1.000	0.000	No significant interaction
Phosphaturia	0.867	0.927	0.060	0.850	0.923	0.073	0.166	0.685	0.003	No significant interaction

Supplementary Table S2. Mixed-design ANOVA results.

Variable	F Group (1,58)	p-value	Partial η²	F Time (1,59)	p-value	Partial η²	F Interaction (1,58)	p-value	Partial η²
NRS	2.930	0.092	0.048	246.991	<0.001	0.807	3.388	0.075	0.054
25(OH)D	0.031	0.862	0.001	198.918	<0.001	0.771	0.000	0.987	0.000
Calcium	0.324	0.571	0.006	149.308	<0.001	0.717	8.193	0.006	0.124
Phosphorus	0.155	0.695	0.003	66.114	<0.001	0.528	3.351	0.072	0.055
Magnesium	0.111	0.741	0.002	52.460	<0.001	0.471	9.201	0.004	0.137
CTX	0.260	0.612	0.005	118.423	<0.001	0.668	7.830	0.007	0.119
ALP	0.021	0.887	0.000	58.531	<0.001	0.498	0.233	0.631	0.004
BALP	3.169	0.080	0.052	84.216	<0.001	0.588	64.721	<0.001	0.527

Variable	F Group (1,58)	p-value	Partial η^2	F Time (1,59)	p-value	Partial η^2	F Interaction (1,58)	p-value	Partial η^2
Calciuria	0.000	0.984	0.000	32.968	<0.001	0.359	0.000	1.000	0.000
Phosphaturia	0.034	0.854	0.001	16.857	<0.001	0.222	0.166	0.685	0.003

Supplementary Table S3. Effect sizes and confidence intervals for significant outcomes.

Outcome	Mean Difference at T1	95% Confidence Interval	Cohen's d	Partial η^2	Interpretation
NRS (pain)	−0.57	−0.98 to −0.16	0.71	0.054	Moderate effect
BALP (U/L)	+5.13	2.48 to 7.79	1.00	0.527	Large effect
Calcium	—	—	—	0.124	Moderate interaction effect
Magnesium	—	—	—	0.137	Moderate interaction effect
CTX	—	—	—	0.119	Moderate interaction effect

Abbreviations: NRS, Numeric Rating Scale; BALP, bone alkaline phosphatase; CTX, C-terminal telopeptide of type I collagen; CI, confidence interval; η^2, partial eta squared.
